# Detection of B.1.351 SARS-CoV-2 Variant Strain — Zambia, December 2020

**DOI:** 10.15585/mmwr.mm7008e2

**Published:** 2021-02-26

**Authors:** Mulenga Mwenda, Ngonda Saasa, Nyambe Sinyange, George Busby, Peter J. Chipimo, Jason Hendry, Otridah Kapona, Samuel Yingst, Jonas Z. Hines, Peter Minchella, Edgar Simulundu, Katendi Changula, King Shimumbo Nalubamba, Hirofumi Sawa, Masahiro Kajihara, Junya Yamagishi, Muzala Kapin’a, Nathan Kapata, Sombo Fwoloshi, Paul Zulu, Lloyd B. Mulenga, Simon Agolory, Victor Mukonka, Daniel J. Bridges

**Affiliations:** ^1^PATH, Lusaka, Zambia; ^2^School of Veterinary Medicine, University of Zambia, Lusaka, Zambia; ^3^Zambia National Public Health Institute, Lusaka, Zambia; ^4^The Wellcome Centre for Human Genetics, Oxford University, Oxford, England; ^5^CDC, Lusaka, Zambia; ^6^Research Center for Zoonosis Control, Hokkaido University, Sapporo, Japan; ^7^Ministry of Health, Lusaka, Zambia.

The first laboratory-confirmed cases of coronavirus disease 2019 (COVID-19), the illness caused by SARS-CoV-2, in Zambia were detected in March 2020 (*1*). Beginning in July, the number of confirmed cases began to increase rapidly, first peaking during July–August, and then declining in September and October (Figure). After 3 months of relatively low case counts, COVID-19 cases began rapidly rising throughout the country in mid-December. On December 18, 2020, South Africa published the genome of a SARS-CoV-2 variant strain with several mutations that affect the spike protein (*2*). The variant included a mutation (N501Y) associated with increased transmissibility.^†^^,^^§^ SARS-CoV-2 lineages with this mutation have rapidly expanded geographically.^¶^^,^** The variant strain (PANGO [Phylogenetic Assignment of Named Global Outbreak] lineage B.1.351^††^) was first detected in the Eastern Cape Province of South Africa from specimens collected in early August, spread within South Africa, and appears to have displaced the majority of other SARS-CoV-2 lineages circulating in that country (*2*). As of January 10, 2021, eight countries had reported cases with the B.1.351 variant. In Zambia, the average number of daily confirmed COVID-19 cases increased 16-fold, from 44 cases during December 1–10 to 700 during January 1–10, after detection of the B.1.351 variant in specimens collected during December 16–23. Zambia is a southern African country that shares substantial commerce and tourism linkages with South Africa, which might have contributed to the transmission of the B.1.351 variant between the two countries.

**FIGURE:**
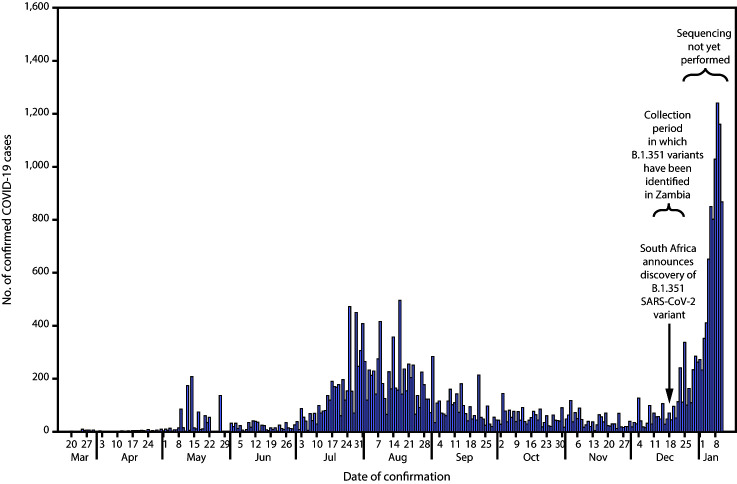
Reported laboratory-confirmed COVID-19 cases, by date of confirmation — Zambia, March 20, 2020–January 11, 2021 **Abbreviation:** COVID-19 = coronavirus disease 2019.

Since September 2020, University of Zambia and PATH (https://www.path.org) have routinely been conducting genetic epidemiologic studies using whole genome sequencing (WGS) on SARS-CoV-2–positive specimens. A subset of specimens collected during March 18–December 23, 2020, were sequenced, from which 268 high-quality genomes were generated. Specimens were selected for WGS based on availability and real-time reverse transcription–polymerase chain reaction (RT-PCR) diagnostic test cycle threshold (Ct) values of <30; lower Ct values are correlated with larger amounts of virus in the sample. Sequences were linked to case investigation information including patient age, sex, and geographic location from routine public health data maintained by the Zambia National Public Health Institute. For WGS, complementary DNA was prepared using random primers from viral RNA extracted from SARS-CoV-2 real-time RT-PCR–positive specimens. Multiplex PCR was then performed using custom primers (*3*) to generate overlapping amplicons for nanopore sequencing on a MinION (Oxford Nanopore Technology, United Kingdom).^§§^ Consensus sequence reads were generated using the standard ARTIC Network bioinformatic pipeline,^¶¶^ a system for processing samples from viral disease outbreaks to generate real-time, actionable epidemiologic information.

Among the 23 specimens collected during December 16–23, 22 (96%) were the B.1.351 variant. None of the 245 previously sequenced genomes was from this lineage. Among the 22 specimens containing the variant strain, 21 (95%) contained all nine B.1.351 lineage-defining mutations. Thirteen (57%) were from males, and the median patient age was 32 years (interquartile range = 27–45 years). Specimens with the B.1.351 variant were obtained from persons in four districts (Lusaka, 16; Livingstone, four; Chingola, one; and Chibombo, one) across four provinces (Lusaka, Southern, Copperbelt, and Central). Five (23%) specimens were obtained from persons in two different clusters, with no known epidemiologic links among other cases.

Detection of the B.1.351 variant coincided with a rapid rise in confirmed cases in Zambia. This detection establishes an epidemiologic linkage between COVID-19 outbreaks in Zambia and South Africa. Spread of the B.1.351 variant is of public health concern because of the potential for increased transmissibility and, thus, increases in cases, hospitalizations, and deaths.*** The B.1.351 variant might be associated with higher viral loads and contains another spike protein mutation (E484K) that might hinder antibody binding,^†††^^,^^§§§^ which could blunt naturally developed immunity or reduce vaccine efficacy. The predominance of the B.1.351 variant in a small cohort of recent specimens suggests that it might have become the dominant lineage in Zambia, although additional WGS of specimens from other districts is needed to characterize the full extent of its spread. Further, the available genomic data could not identify when and from where the B.1.351 variant was introduced to Zambia. Because the B.1.351 variant has been detected in Zambia, it might be circulating elsewhere in southern Africa, where many countries reported rapid increases in numbers of COVID-19 cases during December 2020–January 2021.^¶¶¶^ Phylogenetic analysis and additional sequencing are ongoing to better understand the origin, prevalence, and transmission characteristics of this lineage in Zambia. Expanding capacity for genetic epidemiology in Africa will help strengthen surveillance for the B.1.351 variant as well as early detection of emerging variants that might affect the implementation of vaccination programs.

## Footnotes

†
https://khub.net/documents/135939561/338928724/SARS-CoV-2%20variant%20under%20investigation,%20meeting%20minutes.pdf/962e866b-161f-2fd5-1030-32b6ab467896?t = 1608470511452

§
https://virological.org/t/mutations-arising-in-sars-cov-2-spike-on-sustained-human-to-human-transmission-and-human-to-animal-passage/578

¶
https://cmmid.github.io/topics/covid19/uk-novel-variant.html

**
https://www.who.int/csr/don/31-december-2020-sars-cov2-variants/en/

††
https://github.com/cov-lineages/pangolin/

§§
https://www.protocols.io/view/ncov-2019-sequencing-protocol-v3-locost-bh42j8ye

¶¶
https://artic.network/ncov-2019/ncov2019-bioinformatics-sop.html

***
https://www.cdc.gov/coronavirus/2019-ncov/transmission/variant.html

†††
https://www.biorxiv.org/content/10.1101/2020.12.31.425021v1.full.pdf

§§§
https://www.biorxiv.org/content/10.1101/2020.12.28.424451v1.full.pdf

¶¶¶
https://africacdc.org/download/outbreak-brief-52-coronavirus-disease-2019-covid-19-pandemic/
